# Surveillance of antimicrobial resistance in neonatal intensive care unit of Alexandria University Children’s Hospital

**DOI:** 10.1038/s41598-026-65490-9

**Published:** 2026-08-11

**Authors:** Ehsan Akram Deghidy, Mohamed Alaa El-Din Thabet, Nesrine Fathi Hanafi, Samah Mohamed Shehata, Marwa Mohamed Farag

**Affiliations:** 1 Biomedical Informatics and Medical Statistics Department, Medical Research Institute, Alexandria University, 111 Elrasfa, Moharram Bek, Alexandria, Egypt; 2 Pediatrics and Neonatology, Pediatric Department, Alexandria University Faculty of Medicine, Alexandria, Egypt; 3 Medical Microbiology and Immunology Department, Alexandria University Faculty of Medicine, Alexandria, Egypt

**Keywords:** Neonatal sepsis, WHONET, Klebsiella, Drug-resistant microorganisms, Health care, Medical research, Risk factors

## Abstract

Antimicrobial drugs are fundamental to modern medicine. The rise and spread of drug-resistant microorganisms jeopardize our ability to manage common infections and perform critical medical interventions. The present work aimed to assess and analyze antimicrobial resistance and sensitivity in the neonatal intensive care unit of Alexandria University Children’s Hospital to improve antibiotic choices in the neonatal intensive care unit. A medical record-based study of 1105 neonates with sepsis admitted to the NICU during the four-year study period (2019 to 2022) was conducted, with no exclusion criteria. The data were uploaded to the WHONET system after being collected. The results can be recorded as interpreted values: “R” (Resistant), “I” (Intermediate), and “S” (Sensitive). Interpretation guidelines for most standardized testing methodologies were built into the system. In addition, the program also permits retrieval and correction of clinical records. Among the 1105 neonates included in our study, Male patients accounted for 56.4%, with a mean gestational age of 32.67 ± 2.86 weeks. Early-onset neonatal sepsis (EOS) was detected in 329(29.7%), while late-onset neonatal sepsis (LOS) was detected in 776(70.2%) neonates. Klebsiella was the predominant pathogen, accounting for 554 (50.1%). This organism was responsible for 136(24.5%) neonates in 2019, 148(26.7%) neonates in 2020, 137(24.7%) neonates in 2021 and 133(24.0%) neonates in 2022. Candida species was the second most common pathogen in our NICU; 126 (11.4%) cases were distributed as follows: 31 (24.6%), 32 (25.4%), and 40 (31.7%). 23 (18.2%) cases occurred in 2019, 2020, 2021, and 2022, respectively. Across the 4 years of the study, 2020 had the highest sepsis frequency. Drug resistance was high in most of the isolates. It was maximum (94–80%) for Cefotaxime, Piperacillin, Amoxicillin/Clavulanic acid, and ceftriaxone. It varied between 60 and 80% for Piperacillin/Tazobactam, Imipenem, Ceftazidime, Cefoperazone/Sulbactam, Meropenem, Ampicillin/Sulbactam, and Ciprofloxacin. The current study found that neonatal sepsis had a nosocomial infection profile shared by both early-onset and late-onset sepsis. K. pneumoniae was the predominant pathogen in both EOS and LOS in our study, similar to other developing countries, and is totally different from developed countries.

**Supplementary Information:**

The online version contains supplementary material available at 10.1038/s41598-026-65490-9.

## Introduction

One of the major global health issues is neonatal sepsis. One to ten per thousand live births is the prevalence worldwide. Sepsis is more common in developing nations. For newborns who do not receive treatment, the mortality rate from sepsis might exceed 50%^1^.

Multidrug-resistant (MDR) bacteria that cause neonatal sepsis are frequently associated with a higher mortality rate. Sepsis linked to MDR microbes is a serious issue, particularly in underdeveloped nations, due to the scarcity of treatment options and the high fatality rate^2,3^.

Early diagnosis is the most important element in lowering neonatal sepsis mortality because it allows for the prompt beginning of appropriate therapy. Systems for blood culture have significantly improved sensitivity and reduced detection times. The reduction of mortality and morbidity among neonates with sepsis necessitates improved diagnosis and ongoing evaluation of the effectiveness of the administered treatment^4^.

Multidrug-resistant organisms (MDROs) pose one of the most serious challenges in healthcare-associated and community-acquired infections. MDROs nowadays include Gram-positive and Gram-negative bacteria, as well as atypical organisms that develop resistance^5^.

Surveillance of antimicrobial resistance is fundamental to controlling it and is considered an integral part of any strategy to contain it. It gives information required to locate an antimicrobial problem, monitor its growth and transmission, and determine the impact of intervention^4^.

The World Health Organization (WHO) has established and implemented a surveillance tool for antimicrobial resistance known as WHONET^6^. WHONET analyses culture-sensitivity data very accurately and quickly, making it straightforward to report antibiotic resistance trends in the hospital. This will assist doctors in choosing the right treatment plan and pathogen-directed strategy^7^.

It is a freely available computer program that provides suggestions for empirical infectious treatment and organizes and analyses laboratory results on a personal or global scale.

WHONET gathers all information regarding the test for microbiological culture sensitivity. It gathers all relevant data, including antibiotic names, patient information, and resistance profiles. As a result, a significant amount of data is gathered in one location, which will aid data analysis and serve as a valuable tool for the World Health Organization (WHO) and other governmental organizations in developing antibiotic policies and plans to reduce antibiotic resistance^6^.

Although the growing problem of antimicrobial resistance (AMR) is well understood, comprehensive monitoring and control are hampered by limited resources, a lack of standardized national policies, a shortage of trained personnel, and gaps in data collection and reporting. Understanding local AMR epidemiology and directing successful treatment methods require implementing more systematic hospital surveillance and adopting user-friendly IT technologies such as WHONET.

The present work aimed to assess and analyze antimicrobial resistance and sensitivity in the neonatal intensive care unit of Alexandria University Children’s Hospital to improve antibiotic choices.

## Methods

### Study design, setting, and participants

A medical record-based study was conducted in the Neonatal Intensive Care Unit of Alexandria University Children’s Hospital, Egypt. The data were collected retrospectively over 4 years from January 2019 to December 2022. The data were extracted from 1105 patients’ files to obtain vital information, including test findings, clinical outcomes, symptoms, delivery history, infection risk factors, and demographics. The attending physician gathered the necessary information upon admission. The results of the investigations were collected from the laboratory records using a standard form, and the information was then entered into an Excel spreadsheet.

Following collection, the data was transferred to the WHONET system. Results of susceptibility tests conducted by disk diffusion, MIC, and/or E test can be routinely entered into the application. Interpreted values such as “R” (Resistant), “I” (Intermediate), and “S” (Sensitive) can be used to record the results. The system included interpretation instructions for the majority of standardized testing procedures. Additionally, the application allows healthcare records to be retrieved and corrected.

### The unit protocol for culture withdrawal

Samples were collected for culture and sensitivity testing from every infant who met the study’s inclusion criteria. The blood samples were collected by experienced medical professionals who had received training on the proper technique for acquiring a blood culture specimen. 1–2 mL of blood was collected from a suitable peripheral vein, following strict aseptic precautions using 10% iodine followed by 70% ethanol. The specimens were then immediately transported to the microbiology laboratory.

Cerebrospinal fluid (CSF) by lumbar puncture and urine culture were collected only when LOS was suspected. Chest radiography and swab cultures for superficial infections (umbilical sepsis, pyoderma, etc.) were performed in relevant cases. The isolates were identified by studying colony morphology, Gram staining, and conventional biochemical methods.

### Data analysis

WHONET was used to analyze stored data. The program had a modular configuration that allowed customization of software for clinical, epidemiological, and infection-control applications. From a single screen, a WHONET user can select the type of analysis to run, the species of bacteria to analyze, the subsets of isolates to include [e.g., all, isolates from urine only, and isolates resistant to gentamicin and from certain locations], and the antimicrobial agents and period to examine.

### Statistical analysis

Following coding, data were entered into the Statistical Package for the Social Sciences [IBM-SPSS] for descriptive statistics. The results were presented in diagrams and tables, then interpreted. For quantitative variables, the mean, median, and standard deviation were used; for categorical variables, frequencies and percentages were used.

## Results

### Baseline characteristics, enrollment, and in-hospital course

The baseline characteristics of newborns with neonatal septicemia are depicted in (Table 1). There were 1105 neonatal sepsis cases admitted to the neonatal intensive care unit of Alexandria University Children’s Hospital over 4 years from January 2019 to December 2022. Out of them, 623(56.4%) were male and 482(43.6%) were female. Among our participants, there were 329(29.8%) infants with EOS, 776(70.2%) with LOS. Females and males among neonates with EOS were 36.8% and 63.2%, respectively, and among neonates with LOS were 46.5% and 53.5%, respectively. The mean birth weight among neonates with EOS was (1594.04 ± 837.15 g), ranging from (600 to 4400 g), and among the LOS cases was (1468.50 ± 707 g), ranging from (600 to 4400 g). The mean length of NICU stay was (19.11 ± 15 days and 39.84 ± 24 days) for the EOS and the LOS cases, respectively.

**Table 1:** Baseline characteristics of the studied neonates from 2019 to 2022 in EOS and LOS.

	All cases (No. = 1105)		Study groups				
			Early onset sepsis (No. = 329)		Late onset sepsis (No. = = 776)		
	No.	%	No.	%	No.	%	
Sex							
Female	482	43.6	121	36.8	361	46.5	
Male	623	56.4	208	63.2	415	53.5	
Mode of delivery							
Normal vaginal delivery (NVD)	263	23.8	82	24.9	181	23.3	
Cesarean section (CS)	842	76.2	247	75.1	595	76.7	
Gestational age(weeks)							
28–31^6–7^ weeks	553	50.0	162	49.2	391	50.4	
32^0–7^–33^6–7^ weeks	296	26.7	82	24.9	214	27.6	
34^0–7^–36^6–7^ weeks	151	13.6	47	14.3	104	13.4	
37^0–7^–41 weeks	105	9.5	38	11.6	67	8.6	
Mean ± SD	32.67 ± 2.860		32.71 ± 3.104		32.65 ± 2.752		
(Min–Max)	(28–40)		(28–40)		(28–40)		
Birth weight (grams)							
< 1000	219	19.8	58	17.6	161		20.7
1000– < 1500	589	53.3	169	51.4	420		54.1
1500– < 2500	164	14.8	48	14.6	116		14.9
2500–4000	116	10.5	49	14.9	67		8.6
> 4000	17	1.5	5	1.5	12		1.5
Mean ± SD	1505.88 ± 750.462		1594.04 ± 837.151		1468.50 ± 707.795		
(Min-max)	(600–4400)		(600–4400)		(600–4400)		
1-min Apgar scores							
Mean ± SD	5 ± 1.401		5 ± 1.417		5 ± 1.395		
(Min–max)	(0–7)		(0–7)		(0–7)		
5-min Apgar scores							
Mean ± SD	7 ± 1.521		7 ± 1.429		7 ± 1.559		
(Min -max)	(0–9)		(0–9)		(0–7)		
Length of NICU stay(days)							
Mean ± SD	33.6 ± 23.9		19.11 ± 15.783		39.84 ± 24.196		
(Min–max)	(1–150)		(1–75)		(6–150)		

**Fig. 1:**
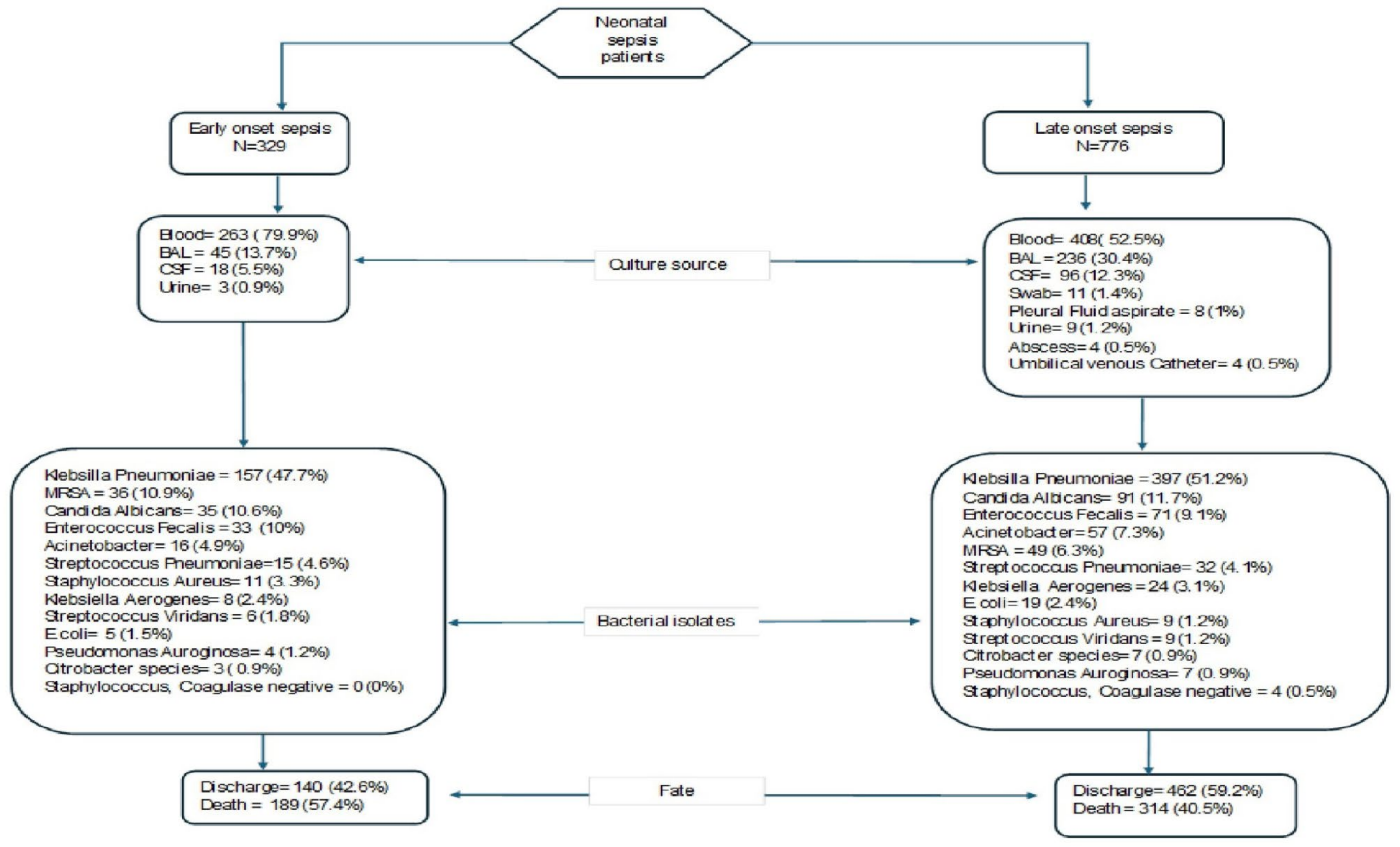
Flow chart of neonates with early and late onset sepsis through 2019 to 2022 in the NICU of Alexandria University Children’s Hospital.

**Fig. 2:**
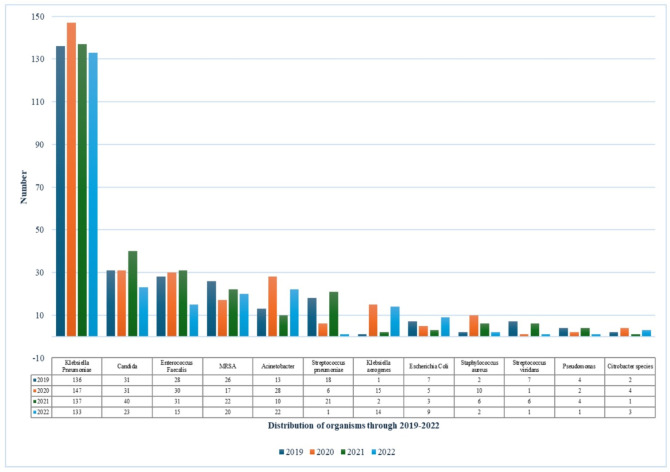
Distribution of organisms according to culture types through 2019 to 2022.

Figure 1 shows a flowchart of neonates with early- and late-onset sepsis. The EOS fatality rate was 57.4% (189 of 329 patients), whereas the LOS fatality rate was 40.5% (314 of 776 patients). Figure 2 shows the distribution of organisms from 2019 to 2022. Among 1105 neonates recruited, Klebsiella was the predominant pathogen, accounting for 554 (50.1%). This organism was responsible for 136 (24.5%) neonates in 2019, 148 (26.7%) in 2020, 137 (24.7%) in 2021, and 133 (24.0%) in 2022. Candida species was the second most common pathogen in our NICU (126 cases; 11.4%), distributed as follows: 31 (24.6%), 32 (25.4%), and 40 (31.7%). 23 (18.2%) neonates during 2019, 2020, 2021, and 2022, respectively.

### Classification of organisms by drug resistance

Among gram-negative bacteria, Klebsiella is the most frequently identified organism in positive cultures. Pan-drug-resistant Klebsiella is observed in 22.5% of cases, while the majority, 70.1%, exhibit extended drug resistance, with susceptibility typically directed towards colistin, doxycycline, Tigecycline, fosfomycin, sulfa drugs, and amikacin. Additionally, 6.4% of cases involve multidrug-resistant Klebsiella.

In contrast, among gram-positive organisms, Enterococcus faecalis is the most prevalent, with 90% of patients harboring multidrug-resistant strains and 10% harboring extended-spectrum-resistant strains.

### Distribution of organisms by gestational age and birth weight

Klebsiella was mostly prominent in the 28–31weeks gestational age group, 287 (51.9%) cases from a total of 553 cases in this gestational age group. The gestational age group 28–31 weeks was the most common age group to be affected by sepsis, 553 (50%) cases. (Table 2).

**Table 2:** Distribution of organisms by gestational age (weeks).

Type of organisms	Gestational age (weeks)							
	28–31		32–33		34–36		37–41	
	No.	%	No.	%	No.	%	No.	%
Klebsiella pneumoniae	287	51.9	146	49.3	70	46.4	51	48.6
Candida	57	10.3	37	12.5	18	11.9	14	13.3
Enterococcus Faecalis	50	9.0	25	8.4	17	11.3	12	11.4
MRSA	45	8.1	24	8.1	9	6	7	6.7
Acinetobacter	40	7.2	15	5.1	10	6.6	8	7.6
Streptococcus pneumoniae	22	4.0	9	3.0	13	8.6	3	2.9
Klebsiella aerogenes	17	3.1	13	4.4	2	1.3	0	0
Escherichia Coli	9	1.6	8	2.7	3	2	4	3.8
Staphylococcus aureus	10	1.8	7	2.4	2	1.3	1	1
Streptococcus viridans	5	0.9	4	1.4	4	2.6	2	1.9
Pseudomonas	3	0.5	5	1.7	1	0.7	2	1.9
Citrobacter species	5	0.9	2	0.7	2	1.3	1	1
Staphylococcus, Coagulase negative	3	0.5	1	0.3	0	0	0	0
*Total*	553	100	296	100	151	100	105	100

Among birthweight groups, the most affected by sepsis was (1000–1500), 589 (53.3%) cases. In this birth weight group, Klebsiella pneumoniae was the most common organism isolated, 295 (50.1%) cases. (Table 3).

**Table 3:** Distribution of organisms by birth weight.

Organisms	Birth weight (grams)									
	< 1000		1000–< 1500		1500–< 2500		2500–4000		> 4000	
	No.	%	No.	%	No.	%	No.	%	No.	%
Klebsiella pneumonia	119	54.3	295	50.1	74	45.1	57	49.1	9	52.9
Candida	21	9.6	73	12.4	18	11	10	8.6	4	23.5
Enterococcus Faecalis	28	12.8	44	7.5	16	9.8	15	12.9	1	5.9
MRSA	14	6.4	46	7.8	15	9.1	10	8.6	0	0
Acinetobacter	14	6.4	39	6.6	10	6.1	10	8.6	0	0
Streptococcus pneumoniae	8	3.7	24	4.1	11	6.7	3	2.6	1	5.9
Klebsiella aerogenes	4	1.8	20	3.4	8	4.9	0	0	0	0
Escherichia Coli	1	0.5	15	2.5	3	1.8	3	2.6	2	11.8
Staphylococcus aureus	4	1.8	11	1.9	3	1.8	2	1.7	0	0
Streptococcus viridans	1	0.5	7	1.2	4	2.4	3	2.6	0	0
Pseudomonas	2	0.9	6	1.0	1	0.6	2	1.7	0	0
Citrobacter species	3	1.4	5	0.8	1	0.6	1	0.9	0	0
Staphylococcus, Coagulase negative	0	0	4	0.7	0	0	0	0	0	0
*Total*	219	100	589	100	164	100	116	100	17	100

### Different antibiotic sensitivities of 5 main families using WHONET program

#### Klebsiella pneumoniae (554 isolates)

**Fig. 3:**
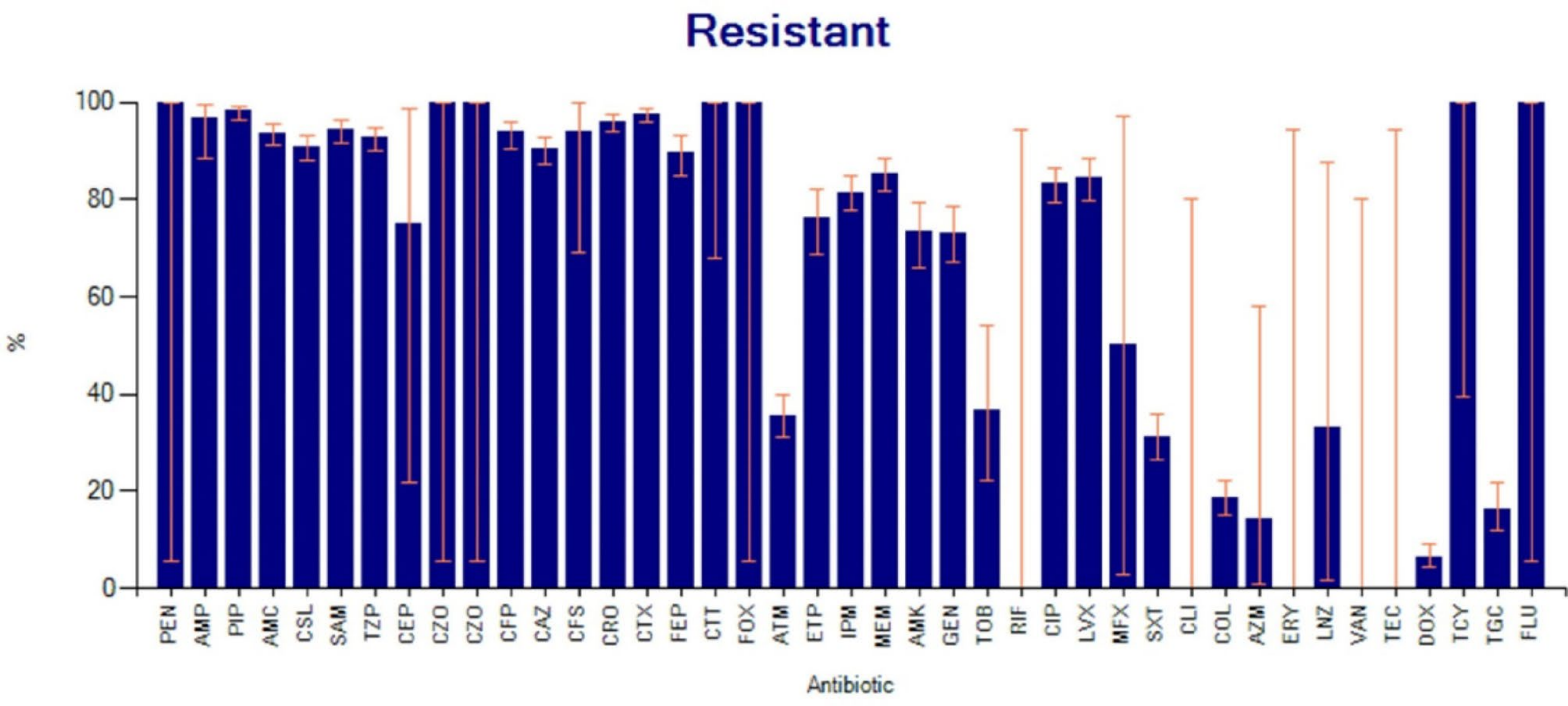
Resistance of Klebsiella to different antibiotics.

Klebsiella pneumoniae, the most common organism recorded in our NICU department and the predominant Gram-negative bacterium isolated in this study. The highest resistance (100%) to cefazolin, Penicillin G, cefoxitin, and tetracycline, followed by Piperacillin (98.3%) and Cefotaxime (97.6%). Moreover, it showed the highest intermediate sensitivity to Erythromycin (100%), followed by Tigecycline (57%) and Moxifloxacin (50%). (Fig. 3).

#### Candida species (126 isolates)

The second recorded organism isolated was Candida species. Candida species resistance to Amphotericin B was 5%, followed by Voriconazole at 3.09%, and lastly, Fluconazole at 2.06%.

#### Enterococcus faecalis (104 isolates)

Enterococcus faecalis was the 3rd most common organism isolated in our study. 100% sensitive to linezolid and trimethoprim/ sulfamethoxazole, but these antibiotics were not used due to high neurotoxicity complications. No sensitivity was reported to many antibiotics, such as piperacillin, Cefoperazone/sulbactam, gentamicin, and amikacin, which were the first-line empirical antibiotics used in our NICU department. No sensitivity was reported for imipenem or meropenem, which were the second-line treatment.

#### MRSA and Staphylococcus aureus (105 isolates)

MRSA showed 100% sensitivity to Moxifloxacin and Vancomycin, 98% to teicoplanin, 97.6% to linezolid, and 95.4% to Doxycycline. MRSA was 0% sensitive to tobramycin.

#### Acinetobacter (73 isolates)

Acinetobacter, the 5th most common organism in our NICU, showed the highest sensitivity to Doxycycline (80%), followed by Tigecycline (67.5%), then colistin (46.3%). No sensitivity was reported to Cefotaxime. Piperacillin, ciprofloxacin, Cefoperazone/ sulbactam, cefepime, Linezolid, vancomycin, teicoplanin, levofloxacin, and tobramycin.

Detailed WHONET program tables (see Additional file 1), [Table S1–S4, Figs. S2 and S3].

### Antimicrobial resistance patterns of gram-negative and gram-positive isolates

Tables (S1, S2) show the antimicrobial resistance pattern for the isolated microorganisms, performed using different antimicrobial agents collected by the WHONET program. Gram-negative bacteria were markedly resistant to Cefotaxime (93.8%), followed by piperacillin (81.3%) and Amoxicillin/Clavulanic acid (79.8%) (Fig. S1). Gram-positive bacteria were mostly resistant to Penicillin G (64.5%), followed by Erythromycin (44%) and Cefoxitin (33.8%) (Fig. S2).

### Antimicrobial susceptibility patterns of gram-negative and positive isolates:

Tables (S3, S4) show the antimicrobial susceptibility patterns for the Gram-negative and Gram-positive organisms using the WHONET program. Gram-negative bacteria were more sensitive to Doxycycline (67.2%) and Aztreonam (46%), followed by Trimethoprim/ Sulfamethoxazole (43.5%) and Colistin (34.7%) (Fig. S3). Gram-positive bacteria were sensitive to Linezolid (78.3%), followed by Vancomycin (71.2%), Teicoplanin (68.5%), and Doxycycline (54.7%) (Fig. S4).

## Discussion

Antimicrobial resistance is a significant problem in hospitals in developing countries. This retrospective study was carried out to determine the bacterial profile of neonates and the antibiotic sensitivity patterns of the isolated pathogens using the WHONET program, to establish a relevant protocol for empirical administration of antimicrobial therapy in view of available data before the culture sensitivity report is available.

In our study, male newborns accounted for 56.4% of patients, while female patients accounted for 43.6%. Similarly, male gender predominance was identified in other studies; they reported comparable results^8–11^.

Neonatal sepsis in low birth weight (LBW) patients has a 7.3-fold higher risk of developing sepsis than neonates born with normal birth weight (BW). About eighty-seven percent of the patients were extremely, very LBW, and moderately LBW. This finding was comparable with a study conducted in Gondar and Bahir Dar, Ethiopia^12^.

This study demonstrated that 849(76.8%) preterm and 151(13.6%) late preterm neonates admitted to the NICU during the study period suffered neonatal sepsis. This agreed with a study conducted in India, which found that preterm patients accounted for 60.5% of patients^13^.

In our study, the mortality rate was 45.5%. Mortality was higher in early onset sepsis (EOS) than late onset sepsis (LOS), which is comparable to an Egyptian study^9^. Another study conducted in India found that the overall mortality rate was 11%^14^, which was higher in the EOS group.

Among the 1105 neonates, LOS was more frequent (70.2%) than EOS. This was consistent with previous studies from other developing countries, such as the Sudanese study, which reported LOS in 70% of cases, and previous Egyptian studies, which reported comparable proportions of 58.8%^9,10^.

Evidence from low- and middle-income countries (LMICs) indicates that Gram-negative bacteria, particularly those in the Enterobacteriaceae family (Klebsiella pneumoniae, E. coli, and Enterobacter species), are the primary cause of LOS in NICUs. followed by Pseudomonas aeruginosa^15–19^.

According to this study, the majority of bacteria causing newborn sepsis in both EOS and LOS were Klebsiella pneumoniae (50%). According to other Egyptian studies^9,20^ carried out at Cairo University by Salama et al. [31.8%)^9^, Deraz et al.(43.3%)^21^ and Almohammady et al. (45.3%)^22^. Additionally, in an Alexandrian study, Klebsiella pneumoniae was identified as the primary cause of EOS and LOS (54%)^1^. Klebsiella pneumoniae was the most frequently isolated bacterium from blood cultures at Zagazig University (37.5%)^23^ and finally at Sohag University (60%)^10^.

Moreover, Similar findings were reported in an Ethiopian study, in which the main causes of LOS were S. aureus and CoNs, while 44% of the samples obtained in EOS were Klebsiella species^23^. The results were also consistent with a South African study (42%) and another from Coimbatore, India (48.9%)^24^.

The higher prevalence of Klebsiella species in this study may be explained by their widespread presence in the hospital environment and their ability to cause infection at birth or during hospitalization^25^. In our study, Klebsiella isolates showed 100% sensitivity to Clindamycin, Vancomycin, Teicoplanin, and Rifampin, 91.2% sensitivity to Doxycycline, and 85.7% sensitivity to Azithromycin, while showing resistance to penicillin.

In our findings, Klebsiella showed 39.6% sensitivity to colistin, 26.8% sensitivity to Tigecycline, and low susceptibility to levofloxacin, ciprofloxacin, amikacin, and gentamicin (14.3%, 10.4%, 21.7%, and 24.8%, respectively). Almohammady et al. found that Klebsiella showed 53% susceptibility to levofloxacin and low susceptibility to ciprofloxacin, amikacin, and gentamicin (12%, 12%, and 6%, respectively)^12,22^.

Moreover, our results showed 90–97% resistance to 3rd-generation cephalosporins, which was consistent with those of Almohammady et al.^22^, in which Klebsiella showed 100% resistance to 3rd-generation cephalosporins.

Regarding Pneumonia, our study found a similar susceptibility profile to that reported in another study conducted in Alexandria, Egypt^1^. A high resistance rate was observed among K. Pneumoniae isolates, including 100% for cephalosporins and tetracyclines, 98.3% for piperacillin, and 97.6% for Cefotaxime. Nevertheless, vancomycin, Erythromycin, clindamycin, and Rifampin were not reported to be resistant to these antibiotics. However, these antibiotics were tested only once or twice against Klebsiella in our study. Regarding the more frequent tests against Klebsiella, the lowest resistance was reported to colistin (18.4%) and Tigecycline (16.2%).

Candida species is the second leading cause of newborn sepsis (11.3%) in both EOS and LOS in the current study. This differs from earlier research reported from Egypt (5.3%)^26^. However, a higher incidence was reported in India: a study reported isolating 32 (9.9%) Candida isolates from 322 positive cases^27^.

Enterococcus faecalis, the most common Gram-positive isolate, accounted for 40.9% of Gram-positive bacteria in the current study. It was almost equally distributed between EOS and LOS (10%).

Enterococcus faecalis accounted for 33% of the Gram-positive bacteria isolated in another study conducted across 3 main referral hospitals in Alexandria^1^. Enterococcus faecalis showed resistance to penicillins, cephalosporins, and quinolones, largely due to the misuse of antibiotics in hospitals, which drives this significant resistance.

In contrast, other studies reported the predominance of Gram-positive bacteria (81.3%) among pathogens causing EOS isolated from a NICU in Tanzania^28^ and another study from King Abdulaziz University Hospital, Jeddah, Saudi Arabia (67.5%)^29^. These inconsistent results might be due to the etiological agents of EOS and LOS changing over time and across geographical locations^8^.

According to an Ethiopian study, the most prevalent infections causing newborn sepsis include GBS, S. aureus, and E. coli.^30^ Another study in southeast Ethiopia found CoNs, E.coli, and S. aureus to be the common causes of neonatal sepsis^31^.

Molecular techniques provide more accurate identification of *Acinetobacter* species and enable better characterization of antimicrobial resistance and virulence genes than conventional phenotypic methods. Future studies should incorporate molecular confirmation to strengthen antimicrobial resistance surveillance^32^.

Generally, increasing antibiotic resistance rates of gram positive and gram negative bacteria with increasing and decreasing trends could be attributed to one or more of the following reasons: Microorganisms naturally evolve antimicrobial resistance which is triggered by the selective pressure caused by extensive misuse and misuse of antibiotics, inadequate infection and disease prevention and control in hospitals, limited access to reasonably priced, high-quality medications, vaccinations, and diagnostics, lack of knowledge and awareness, and lack of implementation of regulations that might encourage the development and dissemination of antibiotic resistance^33–35^.

### Limitations of the study

This study was conducted only at a single hospital; however, our NICU department at Alexandria University, which serves 4 government hospitals, indirectly represented many areas.

Another drawback is that the data are currently four years old, and organisms and antibiotic sensitivities may have altered since then.

WHONET program isn’t used in our microbiological laboratory. It couldn’t provide antimicrobial resistance results based on EOS and LOS, gestational age, or birthweight.

Additionally, this was a WHONET-based surveillance program restricted to culture-positive isolates; infants with clinical sepsis but negative cultures were not captured.

The microbiological laboratory was testing different antibiotics each time, and the kits were not usually available to test the same antibiotic when the culture was positive.

Additionally, molecular methods for confirming Acinetobacter species identification were not available during the study period. Therefore, species identification relied on conventional microbiological methods, which may have affected the accuracy of Acinetobacter identification.

### Points of strength

We intend to introduce a new software program to our NICU, which may help us choose better antibiotics over time. Additionally, the range of cultured organisms and patterns of antibiotic susceptibility can be identified through data analysis spanning 4 years. Thus, the development of empirical antibiotic regimens at the local level may be better guided by this knowledge.

## Conclusions

The work showed that neonatal sepsis in low- and middle-income countries has been found to have a nosocomial infection profile shared by both early-onset and late-onset sepsis. Gram-negative bacteria made up the majority of isolates. K. pneumoniae was the predominant pathogen in both EOS and LOS in our study, similar to other developing countries, and is totally different from developed countries. The most sensitive antibiotics for K. pneumoniae were Clindamycin, Vancomycin, Teicoplanin, and Rifampin, followed by Doxycycline and Azithromycin. Finally, Gram-positive bacterial isolates showed less or no resistance to Clindamycin, Linezolid, Teicoplanin, and Vancomycin. Similarly, Gram-negative bacterial isolates were sensitive to Doxycycline, Aztreonam, Colistin, imipenem, and meropenem.

## Supplementary Information

Below is the link to the electronic supplementary material.

Supplementary Material 1

## Data Availability

All data generated or analyzed during this study are included in this article and its supplementary information files data and can be requested from corresponding author.

## Abbreviations

AMB: Amphotericin B
AMC: Amoxicillin/clavulanic acid
AMK: Amikacin
AMP: Ampicillin
AMR: Antimicrobial resistance
APGAR: Appearance, pulse, grimace, activity and respiration
CoNs: Coagulase-negative staphylococci
E. Coli: Escherichia Coli
EOS: Early onset sepsis
K. pneumoniae: Klebsiella pneumoniae
LBW: Low birth weight
LOS: Late onset sepsis
LP: Lumber puncture
LVX: Levofloxacin
NICU: Neonatal intensive care unit
