# Supplementary material for: Surveillance of antimicrobial resistance in neonatal intensive care unit of Alexandria University Children’s Hospital
Source: Sci Rep. 2026 Aug 11;16:24917. doi: 10.1038/s41598-026-65490-9 (PMC13462736; doi:10.1038/s41598-026-65490-9)
Supplement: Supplementary file 1 [file 41598_2026_65490_MOESM1_ESM.docx]

Table S1: Antimicrobial resistance pattern of Gram-negative isolates

| **Antibiotics** | **% Resistance in Gram-negative isolates** | | | | | |
| --- | --- | --- | --- | --- | --- | --- |
|  | ***Klebsiella pneumoniae*** | ***Acinetobacter*** | ***Escherichia Coli*** | ***Pseudomonas aeruginosa*** | ***Citrobacter*** | ***total*** |
|  | ***N= 553*** | ***N= 73*** | ***N= 24*** | ***N= 11*** | ***N= 10*** | ***N= 671*** |
| Cefotaxime | 531 (97.6%) | 66 (97%) | 28 (84.8%) |  | 5 (50%) | 630 (93.8%) |
| Piperacillin | 461 (98.3%) | 56 (100%) | 22 (78.5%) | 2 (20%) | 5 (50%) | 546 (81.3%) |
| Amoxicillin/Clavulanic acid | 477 (93.7% | 36 (80%) | 18 (60%) |  | 5 (50%) | 536 (79.8%) |
| Ceftriaxone | 446 (99.1%) | 64 (96.6%) | 20 (74%) |  | 5 (50%) | 535 (79.7%) |
| Piperacillin/Tazobactam | 448 (92.8%) | 64 (91.4%) | 16 (53.3%) | 0 | 5 (50%) | 533 (79.4%) |
| Imipenem | 437 (81.5%) | 70 (94.5%) | 10 (32.2%) | 0 | 5 (50%) | 522 (77.8%) |
| Ceftazidime | 428 (90.5%) | 64 (96.9%) | 18 (64.2%) | 0 | 5 (50%) | 515 (76.7%) |
| Cefoperazone/Sulbactam | 430 (90.9%) | 38 (90.4%) | 14 (42.4%) |  | 5 (50%) | 487 (72.5%) |
| Meropenem | 390 (85.3%) | 58 (93.5%) | 11 (44%) | 0 | 5 (50%) | 464 (69.1%) |
| Trimethoprim/Sulfamethoxazole | 126 (31%) | 43 (69.3%) | 13 (54.1%) |  |  | 419 (62.4%) |
| Ampicillin/Sulbactam | 364 (94.5) | 36 (80%) | 14 (60.8%) |  | 5 (55.5%) | 419 (62.4%) |
| Ciprofloxacin | 351 (83.2%) | 45 (100%) | 16 (76.1%) | 2 (25%) |  | 414 (61.9%) |
| Cefoperazone | 302 (93.8%) | 37 (94.8%) | 16 (53.3%) | 0 |  | 355 (52.9%) |
| Levofloxacin | 249 (84.7%) | 29 (93.5%) | 11 (57.8%) | 0 |  | 289 (43%) |
| Cefepime | 193 (89.8%) | 16 (94.1%) | 11 (64.7%) | 0 |  | 220 (32.7%) |
| Gentamicin | 186 (73.2%) | 20 (90.9%) | 11 (64.7%) | 0 |  | 217 (32.3%) |
| Amikacin | 132 (73.3%) | 45 (84.9%) | 3 (27.2%) | 0 |  | 180 (26.8%) |
| Aztreonam | 169 (35.4%) |  | 7 (25%) | 0 | 1 (10%) | 177 (26.3%) |
| Ertapenem | 127 (76%) |  | 2 (40%) |  |  | 129 (19.2%) |
| Colistin | 86 (18.4%) | 3 (4.3%) | 0 | 0 | 3 (30%) | 92 (13.7%) |
| Ampicillin | 63 (96.9%) |  | 5 (100%) |  |  | 68 (10.1%) |
| Tigecycline | 38 (16.2%) | 6 (16.2%) | 0 |  |  | 44 (6.5%) |
| Doxycycline | 26 (6.2%) | 4 (7.2%) | 2 (10%) |  |  | 32 (4.7%) |
| Cefsulodin | 16 (94.1%) |  | 2 (100%) |  |  | 18 (2.6%) |
| Tobramycin | 14 (36.8%) | 2 (100%) |  |  |  | 16 (2.3%) |
| Cefotetan | 11 (100%) |  | 1 (100%) |  |  | 12 (1.7%) |
| Tetracycline | 4 (100%) |  |  |  |  | 4 (0.5%) |
| Cephalothin | 3 (75%) |  |  |  |  | 3 (0.4%) |
| Linezolid | 1 (33.3) | 2 (100%) |  |  |  | 3 (0.4%) |
| Vancomycin | 0 | 2 (100%) |  |  |  | 2 (0.29%) |
| Teicoplanin | 0 | 2 (100%) |  |  |  | 2 (0.29%) |
| Azithromycin | 1 (14.3%) |  |  |  |  | 1 (0.1%) |
| Moxifloxacin | 1 (50%) |  |  |  |  | 1 (0.1%) |
| Cefoxitin | 1 (100%) |  |  |  |  | 1 (0.1%) |
| Penicillin G | 1 (100%) |  |  |  |  | 1 (0.1%) |
| Cefazolin | 1 (100%) |  |  |  |  | 1 (0.1%) |
| Clindamycin | 0 |  |  |  |  |  |
| Erythromycin | 0 |  |  |  |  |  |
| Rifampin | 0 |  |  |  |  |  |

B-lactams: cefazolin, moxifloxacin, Penicillin G, Cephalothin, Cefotetan, Ampicillin, Cefepime, Amoxicillin/Clavulanic acid, Cefotaxime.

Piperacillin, Cefoxitin, Ceftriaxone, Piperacillin/Tazobactam, Ceftazidime, Cefsulodin,Cefoperazone/Sulbactam, Ampicillin/Sulbactam.

Aminoglycosides: Gentamicin, Amikacin, Tobramycin.

Polypeptides: Rifampicin

Quinolones: Ciprofloxacin, Levofloxacin, Ofloxacin

Polyketide: Doxycycline

Glycopeptides: Vancomycin, Teicoplanin

Macrolides: Azithromycin, Erythromycin

Lincomycin derivatives: Clindamycin

Polymyxin: Colistin

Glycylcycline: Tigecycline

Carbapenems: Imipenem and meropenem

**Figure S1: Antimicrobial resistance pattern of Gram-negative isolates**

Table S2: Antimicrobial resistance pattern of Gram-positive isolates.

| **Antibiotics** | **% Resistance in Gram-positive isolates** | | | | | |
| --- | --- | --- | --- | --- | --- | --- |
|  | ***Enterococcus faecalis*** | ***MRSA*** | ***Streptococcus pneumoniae*** | ***Streptococcus viridans*** | ***CONs*** | ***Total*** |
|  | ***N=104*** | ***N= 85*** | ***N= 46*** | ***N= 15*** | ***N= 4*** | ***N=254*** |
| Penicillin G | 67 (87.01%) | 91 (97.8%) |  | 4 (36.6%) | 2 (50%) | 164 (64.5%) |
| Erythromycin | 52 (92.8) | 57 (65.5%) | 2 (9%) | 1 (11.1%) | 0 | 112 (44%) |
| Cefoxitin | 2 (100%) | 84 (80%) |  |  | 0 | 86 (33.8%) |
| Ciprofloxacin | 40 (90.9%) | 40 (48.1%) |  |  | 0 | 80 (31.4%) |
| Gentamicin | 55 (82.08%) | 22 (56.4%) |  |  |  | 77 (30.3%) |
| Trimethoprim/Sulfamethoxazole | 0 | 40 (46.5%) | 30 (83.3%) |  | 0 | 70 (27.5%) |
| Vancomycin | 63 (77.7%) | 0 | 0 | 3 (20%) | 0 | 66 (25.9%) |
| Levofloxacin | 21 (75%) | 24 (53.3%) | 18 (78.2%) | 0 | 0 | 63 (24.8%) |
| Azithromycin |  | 63 (63.6%) |  |  | 0 | 63 (24.8%) |
| Ofloxacin | 4 (100%) | 31 (53.4%) | 16 (80%) | 1 (20%) | 0 | 52 (20.4%) |
| Teicoplanin | 52 (65.8%) | 0 | 0 | 0 | 0 | 52 (20.4%) |
| Clindamycin |  | 43 (46.2%) | 2 (5%) | 6 (54.4%) | 0 | 51 (20%) |
| Ampicillin | 43 (84.3%) |  |  | 4 (44.4%) |  | 47 ((18.5%) |
| Rifampin | 19 86.3%) | 12 (42.8%) | 0 |  | 0 | 31 (12.2%) |
| Doxycycline | 19 (27.1%) | 2 (2.2%) | 8 (30.7%) |  | 0 | 29 (11.4%) |
| Cefotaxime | 13 (100%) |  |  | 7 (53.8%) |  | 20 (7.8%) |
| Ceftriaxone | 11 (100%) |  |  | 7 (63.6%) |  | 18 (7%) |
| Cefoperazone | 12 (100%) |  |  | 2 (40%) |  | 14 (5.5%) |
| Piperacillin | 13 (100%) |  |  | 0 |  | 13 (5.1%) |
| Amoxicillin/Clavulanic acid | 13 (100%) |  |  |  |  | 13 (5.1%) |
| Piperacillin/Tazobactam | 13 (100%) |  |  |  |  | 13 (5.1%) |
| Ceftazidime | 13 (100%) |  |  |  |  | 13 (5.1%) |
| Imipenem | 13 (100%) |  |  |  |  | 13 (5.1%) |
| Meropenem | 13 (100%) |  |  |  |  | 13 (5.1%) |
| Amikacin | 3 (100%) | 10 (25%) |  |  | 0 | 13 (5.1%) |
| Cefoperazone/Sulbactam | 11 (100%) |  |  |  |  | 11 (4.3%) |
| Ampicillin/Sulbactam | 7 (100%) |  |  |  |  | 7 (2.7%) |
| Cefepime | 1 (100%) |  |  | 6 (60%) |  | 7 (2.7%) |
| Linezolid | 0 | 2 (2.3%) | 0 | 2 (14.2%) | 0 | 4 (1.5%) |
| Aztreonam | 2 (15.3%) |  |  |  |  | 2 (0.7%) |
| Colistin | 2 (15.3%) |  |  |  |  | 2 (0.7%) |
| Nitrofurantoin | 2 (50%) |  |  |  |  | 2 (0.7%) |
| Tetracycline |  | 2 (33.3%) |  |  |  | 2 (0.7%) |
| Tobramycin |  | 2 (100%) |  |  | 0 | 2 (0.7%) |
| Cefazolin | 1 (50%) |  |  |  |  | 1 (0.3%) |
| Tigecycline | 0 |  |  |  |  | 0 |
| Clarithromycin |  | 0 |  |  |  | 0 |

**Figure S2: Antimicrobial resistance pattern of Gram-positive isolates**

**Table S3: Antimicrobial susceptibility pattern of Gram-negative isolates.**

| **Antibiotic name** | **% sensitivity in Gram-negative isolates** | | | | | |
| --- | --- | --- | --- | --- | --- | --- |
|  | ***Klebsiella pneumoniae*** | ***Acinetobacter*** | ***Escherichia Coli*** | ***Pseudomonas aeruginosa*** | ***Citrobacter*** | ***Total*** |
|  | ***N= 553*** | ***N= 73*** | ***N= 24*** | ***N= 11*** | ***N= 10*** | ***N=671*** |
| Doxycycline | 384 (91.2%) | 44 (80%) | 16 (80%) |  | 7 (100%) | 451 (67.2%) |
| Aztreonam | 286 (60%) |  | 10 (35.7%) | 6 (60%) | 7 (70%) | 309 (46%) |
| Trimethoprim/Sulfamethoxazole | 271 (66.7%) | 8 (12.9%) | 9 (37.5%) |  | 4 (57.1%) | 292 (43.5%) |
| Colistin | 185 (39.6%) | 32 (46.3%) | 10 (32.2%) | 4 (50%) | 2 (20%) | 233 (34.7%) |
| Imipenem | 64 (11.9%) | 4 (5.4%) | 19 (61.2%) | 12 (100%) | 5 (50%) | 104 (15.5%) |
| Tigecycline | 63 (26.8%) | 25 (67.5%) | 2 (50%) |  | 2 (100%) | 92 (13.7%) |
| Meropenem | 46(10.1%) | 4 (6.4%) | 14 (56%) | 10 (100%) | 5 (50%) | 79 (11.7%) |
| Gentamicin | 63 (24.8%) | 2 (9.09%) | 6 (35.2%) | 4 (100%) | 3 (42.8%) | 78 (11.6%) |
| Ceftazidime | 37 (7.8%) | 2 (3.03%) | 6 (21.4%) | 10 (100%) | 5 (50%) | 60 (8.9%) |
| Piperacillin/Tazobactam | 24 (5%) | 4 (5.7%) | 12 (40%) | 12 (100%) | 5 (50%) | 57 (8.5%) |
| Ciprofloxacin | 44 (10.4%) | 0 | 3 (14.2%) | 6 (75%) | 4 (44.4%) | 57 (8.5%) |
| Levofloxacin | 42 (14.3%) | 2 (6.4%) | 5 (26.3%) | 4 (100%) | 3 (60%) | 56 (8.3%) |
| Amikacin | 39 (21.7%) | 4 (7.5%) | 8 (72.7%) | 4 (100%) | 1 (20%) | 56 (8.3%) |
| Cefoperazone/Sulbactam | 24 (2.1%) | 0 | 14 (42.4%) |  | 5 (50%) | 43 (6.4%) |
| Amoxicillin/Clavulanic acid | 19 (3.7%) | 4 (12.9%) | 12 (40%) |  | 5 (50%) | 40 (5.9%) |
| Ampicillin/Sulbactam | 17 (4.4%) | 6 (13.3%) | 9 (39.1%) |  | 4 (44.4%) | 36 (5.3%) |
| Ceftriaxone | 17 (3.7%) | 2 (3.03%) | 7 (25.9%) |  | 5 (50%) | 31 (4.6%) |
| Cefoperazone | 10 (3.1%) | 2 (5.1%) | 5 (33.3%) | 8 (100%) | 2 (66.6%) | 27 (4%) |
| Ertapenem | 23 (13.8%) |  | 3 (60%) |  | 1 (100%) | 27 (4%) |
| Piperacillin | 7 (1.5%) | 0 | 4 (14.2%) | 8 (80%) | 5 (50%) | 24 (3.5%) |
| Tobramycin | 22 (57.9%) | 0 |  |  |  | 22 (3.2%) |
| Cefotaxime | 9 (1.7%) | 0 | 5 (15.1%) |  | 5 (50%) | 19 (2.8%) |
| Cefepime | 6 (2.8%) | 0 | 3 (17.6%) | 4 (100%) | 3 (42.8%) | 16 (2.3%) |
| Azithromycin | 6 (85.7%) |  |  |  |  | 6 (0.9%) |
| Linezolid | 2 (66.7%) | 0 |  |  |  | 2 (0.3%) |
| Clindamycin | 2 (100%) |  |  |  |  | 2 (0.3%) |
| Vancomycin | 2 (100%) | 0 |  |  |  | 2 (0.3%) |
| Cefsulodin | 1 (5.9%) |  | 0 |  |  | 1 (0.1%) |
| Cephalothin | 1 (25%) |  |  |  |  | 1 (0.1%) |
| Teicoplanin | 1 (100%) | 0 |  |  |  | 1 (0.1%) |
| Rifampin | 1 (100%) |  |  |  |  | 1 (0.1%) |
| Ampicillin | 0 |  | 0 |  | 0 | 0 |
| Cefotetan | 0 |  | 0 |  |  | 0 |
| Tetracycline | 0 |  |  |  |  | 0 |
| Moxifloxacin | 0 |  |  |  |  | 0 |
| Cefoxitin | 0 |  |  |  |  | 0 |
| Penicillin G | 0 |  |  |  |  | 0 |
| Erythromycin | 0 |  |  |  |  | 0 |
| Cefazolin | 0 |  |  |  |  | 0 |
| Lomefloxacin |  | 0 |  |  |  | 0 |

**Figure S3: Antimicrobial susceptibility pattern of Gram-negative isolates**

Table S4: Antimicrobial susceptibility pattern of Gram-positive isolates.

| **Antibiotics** | **% sensitivity in Gram-positive isolates** | | | | | |
| --- | --- | --- | --- | --- | --- | --- |
|  | ***Enterococcus faecalis*** | ***MRSA*** | ***Streptococcus pneumoniae*** | ***Streptococcus viridans*** | ***CONs*** | ***Total*** |
|  | ***N=104*** | ***N= 85*** | ***N= 46*** | ***N= 15*** | ***N= 4*** | ***N=254*** |
| Linezolid | 63 (100%) | 83 (97.6%) | 36 (100%) | 12 (85.7%) | 5 (100%) | 199 (78.3%) |
| Vancomycin | 14 (17.2%) | 105 (100%) | 45 (100%) | 12 (80%) | 5 (100%) | 181 (71.2%) |
| Teicoplanin | 21 (26.5%) | 103 (98.09%) | 1 (100%) | 44 (84.6%) | 5 (100%) | 174 (68.5%) |
| Doxycycline | 39 (55.7%) | 83 (95.4%) | 14 (53.8%) |  | 3 (100%) | 139 (54.7%) |
| Clindamycin |  | 46 (49.6%) | 38 (95%) | 4 (36.3%) | 5 (100%) | 93 (36.6%) |
| Trimethoprim/Sulfamethoxazole | 11 (11%) | 46 (53.4%) | 6 (16.6%) |  | 2 (100%) | 65 (25.5%) |
| Erythromycin | 4 (7.1%) | 26 (29.8%) | 12 (54.5%) | 6 (66.6%) | 5 (100%) | 53 (20.8%) |
| Ciprofloxacin | 2 (4.5%) | 39 (46.9%) |  |  | 2 (100%) | 43 (16.9%) |
| Levofloxacin | 4 (14.2%) | 21 (46.6%) | 4 (17.3%) | 8 (100%) | 3 (100%) | 40 (15.7%) |
| Azithromycin |  | 34 (34.3%) |  |  | 5 (100%) | 39 (15.3%) |
| Ofloxacin | 0 | 27 (46.5%) | 4 (20%) | 2 (40%) | 2 (100%) | 35 (13.7%) |
| Amikacin | 0 | 30 (75%) |  |  | 1 (100%) | 31 (12.2%) |
| Gentamicin | 12 (17.9%) | 17 (43.5%) |  |  |  | 29 (11.4%) |
| Rifampin | 2 (9.09%) | 16 (57.1%) | 8 (100%) |  | 2 (100%) | 28 (11%) |
| Cefoxitin | 0 | 21 (20%) |  |  | 5 (100%) | 26 (10.2%) |
| Penicillin G | 10 (12.9%) | 2 (2.1%) |  | 7 (63.6%) | 5 (100%) | 24 (9.4%) |
| Ampicillin | 8 (15.6%) |  |  | 5 (55.5%) |  | 13 (5.1%) |
| Aztreonam | 11 (84.6%) |  |  |  |  | 11 (4.3%) |
| Colistin | 10 (76.9%) |  |  |  |  | 10 (3.9%) |
| Cefotaxime | 0 |  |  | 4 (30.7%) |  | 4 (1.5%) |
| Cefepime | 0 |  |  | 4 (40%) |  | 4 (1.5%) |
| Tetracycline |  | 4 (66.6%) |  |  |  | 4 (1.5%) |
| Piperacillin | 0 |  |  | 2 (100% |  | 2 (0.7%) |
| Cefoperazone | 0 |  |  | 2 (40%) |  | 2 (0.7%) |
| Ceftriaxone | 0 |  |  | 2 (18.1%) |  | 2 (0.7%) |
| Nitrofurantoin | 2 (50%) |  |  |  |  | 2 (0.7%) |
| Clarithromycin |  | 2 (50%) |  |  |  | 2 (0.7%) |
| Telithromycin |  |  |  | 2 (100%) |  | 2 (0.7%) |
| Cefazolin | 1 (50%) |  |  |  |  | 1 (0.4%) |
| Tobramycin |  | 0 |  |  | 1 (100%) | 1 (0.4%) |
| Moxifloxacin |  | 1 (100%) |  |  |  | 1 (0.4%) |
| Amoxicillin/Clavulanic acid | 0 |  |  |  |  | 0 |
| Cefoperazone/Sulbactam | 0 |  |  |  |  | 0 |
| Ampicillin/Sulbactam | 0 |  |  |  |  | 0 |
| Piperacillin/Tazobactam | 0 |  |  |  |  | 0 |
| Ceftazidime | 0 |  |  |  |  | 0 |
| Imipenem | 0 |  |  |  |  | 0 |
| Meropenem | 0 |  |  |  |  | 0 |
| Tigecycline | 0 |  |  |  |  | 0 |

**Figure S4: Antimicrobial susceptibility pattern of Gram-positive isolates**
